# Distribution of urinary stone composition and its associations with age, sex, and temporal factors among 3901 patients in Xiamen: A 13-year single-center retrospective study

**DOI:** 10.1097/MD.0000000000048826

**Published:** 2026-05-29

**Authors:** Wei Yan, Danfeng Sun

**Affiliations:** a Department of General Surgery (Thyroid and Parathyroid Surgery), Zhongshan Hospital, Xiamen University, Xiamen, Fujian Province, P. R. China; b Department of Urological Surgery, Zhongshan Hospital, Xiamen University, Xiamen, Fujian Province, P. R. China.

**Keywords:** age, Fourier-transform infrared spectroscopy, restricted cubic spline, sex, stone composition, urolithiasis

## Abstract

Regional climate and lifestyle are key drivers of urolithiasis, yet long-term data from subtropical coastal regions in China are limited. This study investigated the epidemiological characteristics and dynamic age-sex associations of stone composition in Xiamen, a major maritime city in Southern China. We analyzed 3901 patients with urolithiasis at a tertiary center between March 2012 and June 2025. Stone components were identified using Fourier-transform infrared spectroscopy. Beyond standard categorical analysis, we employed restricted cubic spline (RCS) models to describe the precise nonlinear associations between age, sex, and the risk of major stone types. Calcium oxalate (CaOx) was the predominant component (73.9%), though mixed stones were remarkably frequent (82.4%). We observed robust sex-specific profiles: men showed higher rates of CaOx and uric acid (UA) stones, whereas women were more prone to calcium phosphate (CaP) and magnesium ammonium phosphate (MgP/infection) stones. RCS analysis revealed distinct risk trajectories: CaOx followed an inverted U-shaped pattern peaking in midlife (45–50 years); CaP risk generally declined with age. Notably, UA risk demonstrated a J-shaped escalation, rising exponentially after age 50, particularly in men. In women, MgP risk followed a U-shaped curve, increasing sharply after age 70. Despite the subtropical setting, no significant seasonal variations were found, suggesting that the stable maritime climate may dampen the thermal drivers of stone formation seen in continental regions. Urinary stone composition in this coastal population follows complex, sex-dependent, and nonlinear age trajectories. The late-life surge in UA stones among men and infection stones among women highlights the need for stratified, age-specific metabolic and clinical management. These findings provide a data-driven framework for personalized urolithiasis prevention in Southern China.

## 1. Introduction

Urolithiasis is a common urological disease worldwide and is characterized by a high recurrence rate and substantial disease burden.^[1]^ A recent meta-analysis estimated the global prevalence of urolithiasis at approximately 10.85%, although considerable geographic variation persists.^[2]^ Such variation is largely related to differences in climate, diet, lifestyle, and population structure.

Stone composition analysis is fundamental to understanding stone-forming mechanisms and developing preventive strategies. Different stone components correspond to distinct pathophysiological backgrounds. Calcium oxalate stones are commonly associated with metabolic abnormalities and urinary supersaturation; uric acid stones are more frequently observed in older individuals and in patients with acidic urine or metabolic syndrome; infection stones are typically associated with urinary tract infection and alkaline urine; and some calcium phosphate (CaP) stones are related to higher urinary pH.^[1–4]^ Therefore, evaluating age, sex, stone location, and temporal patterns according to stone composition has direct clinical relevance.

Previous studies have shown that calcium oxalate remains the predominant stone type, although substantial heterogeneity exists in the distribution of stone composition across age, sex, geographic region, and season.^[3–8]^ Multiple studies in China have suggested that calcium oxalate and uric acid stones are more common in men, whereas carbonate apatite and infection stones are more common in women. Uric acid stones increase with age, whereas some calcium-containing stones are more common in younger populations.^[3,4,9–15]^ However, long-term continuous data from subtropical coastal cities in southern China remain insufficient, and the nonlinear relationship between age and stone type has not been systematically characterized.

Xiamen is located on the southeastern coast of China and has a subtropical maritime monsoon climate characterized by warmth, humidity, and relatively small annual temperature fluctuations. Its dietary patterns and environmental exposures differ from those in northern and inland regions of China. Against this background, we retrospectively analyzed the composition distribution of urinary stones in 3901 patients treated at Zhongshan Hospital, Xiamen University, from March 2012 to June 2025. We assessed the associations of stone composition with sex, age, stone location, and temporal factors, and further used restricted cubic spline (RCS) models to describe the nonlinear association between age and the risk of major stone types.

## 2. Materials and methods

This report follows the Strengthening the Reporting of Observational Studies in Epidemiology guideline for cross-sectional studies.

### 2.1. Study population

This was a single-center retrospective cross-sectional study conducted at Zhongshan Hospital, Xiamen University. The study protocol was approved by the hospital ethics committee (Approval No. 24043110) and was conducted in accordance with the Declaration of Helsinki. The requirement for informed consent was waived because the study was based on anonymized historical medical records and involved no additional intervention.

**Figure 1.**
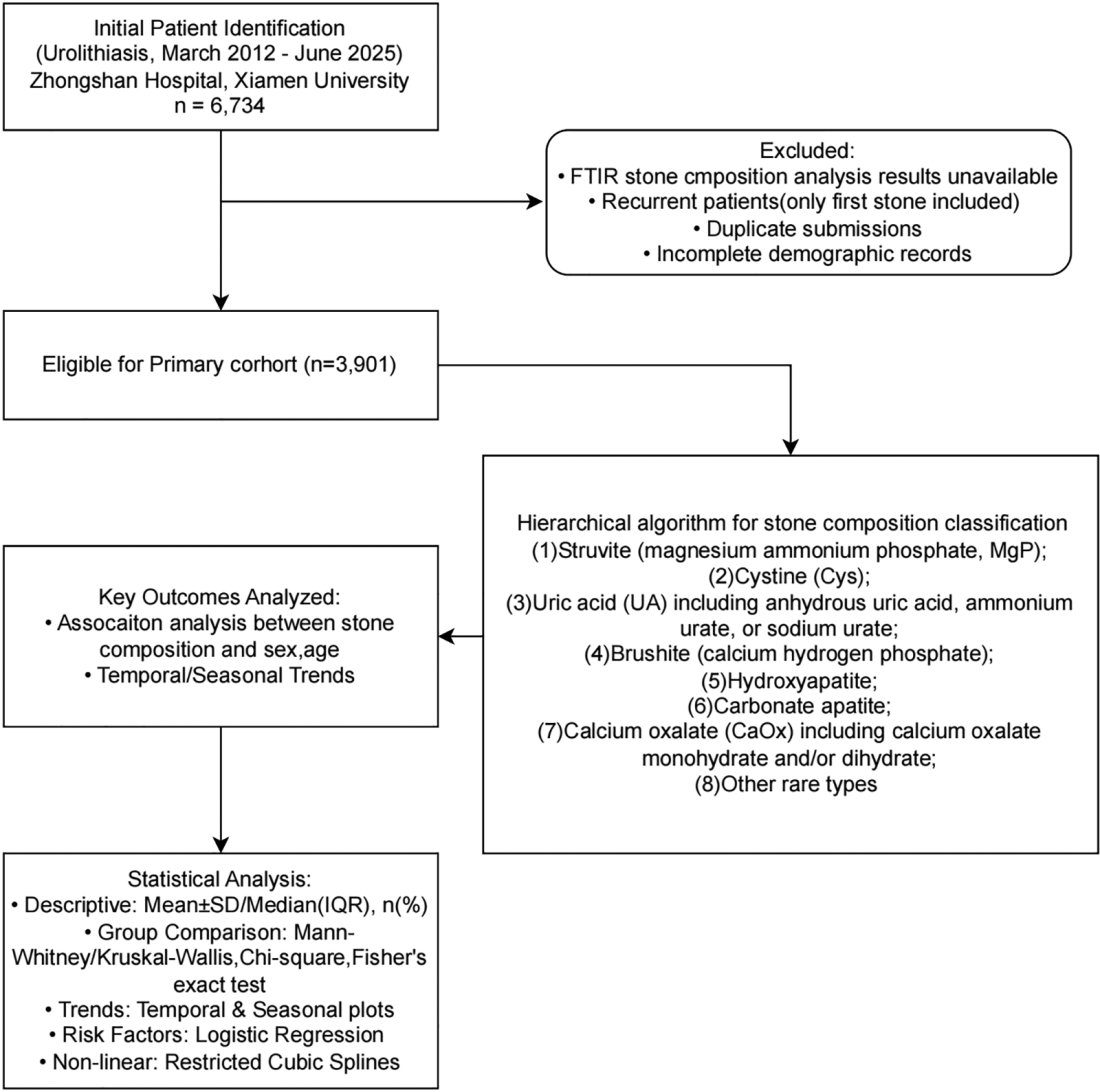
Flowchart of patient selection and study design.

Patients diagnosed with urinary stones and having completed stone composition analysis between March 2012 and June 2025 were eligible for inclusion. All stone patients presented to the hospital for the first time due to symptoms induced by urinary stones, but not all stones were surgically retrieved, as some patients passed stones spontaneously after conservative medical treatment. Surgical procedures included open surgery, ureteroscopy, percutaneous nephrolithotomy, and extracorporeal shock wave lithotripsy (spontaneously passed stones were also collected and analyzed) and so on. The inclusion criteria were as follows: (1) urinary stones confirmed by imaging examinations, including ultrasonography, computed tomography, or plain radiography; (2) available fourier-transform infrared spectroscopy (FTIR-based) stone composition results; and (3) complete core demographic and clinical data. For patients with recurrent stones, only the first submitted stone analysis result was retained to ensure the independence of observations. After excluding cases with missing data or unclear composition classification, 3901 patients were ultimately included in the final analysis (Fig. 1).

### 2.2. Data collection

Data on sex, age, and stone location were extracted from the electronic medical record system. The stone location was determined according to preoperative noncontrast computed tomography or ultrasonography findings and cross-checked with intraoperative observations. Stones were classified as renal, ureteral, bladder, or urethral. Age was analyzed both as a continuous variable and in 10-year strata. For subgroup comparisons, patients were further categorized into 3 clinically relevant age groups: 40 years or younger, 41–60 years, and older than 60 years.

### 2.3. Classification of stone composition

All stone samples were analyzed by FTIR. To ensure mutually exclusive classification, stones were assigned to a single category according to a prespecified hierarchical algorithm^[6]^: (1) stones containing magnesium ammonium phosphate (MgP) were classified as MgP stones; (2) if MgP was absent but cystine (Cys) was present, stones were classified as Cys stones; (3) if neither of the above was present but uric acid-related components were detected, including anhydrous uric acid, ammonium urate, or sodium urate, stones were classified as uric acid (UA) stones; (4) stones containing calcium hydrogen phosphate were classified as brushite; (5) stones containing hydroxyapatite were classified as hydroxyapatite; (6) stones in which carbonate apatite accounted for at least 50% were classified as carbonate apatite; (7) stones in which calcium oxalate monohydrate and/or dihydrate accounted for at least 50% were classified as calcium oxalate (CaOx) stones; and (8) all remaining rare components were grouped as other types. For statistical analysis, brushite, hydroxyapatite, and carbonate apatite were combined and defined as CaP stones.

### 2.4. Statistical analysis

Continuous variables are presented as mean ± standard deviation or median (interquartile range, IQR), as appropriate, and categorical variables are presented as counts and percentages. Between-group comparisons of continuous variables were performed using the Mann–Whitney *U* test or Kruskal–Wallis test, while categorical variables were compared using the chi-square test or Fisher exact test.

Annual trends were described by plotting the proportions of major stone types by calendar year. Monthly analyses compared distributions across January to December. For seasonal analyses, spring was defined as March to May, summer as June to August, autumn as September to November, and winter as December to February of the following year.

Multivariable logistic regression was used to evaluate the independent associations of sex, age, and other factors with the 4 major stone types, namely CaOx, CaP, UA, and MgP. Results are presented as adjusted odds ratios (ORs) and 95% confidence intervals. RCS models were further constructed to explore nonlinear associations between age and the risk of each stone type. Models with 3 to 7 knots were compared, and the optimal model was selected according to the Akaike information criterion. All statistical analyses were performed using R version 4.4.1. A 2-sided *P* value < .05 was considered statistically significant.

## 3. Results

### 3.1. Baseline characteristics

**Figure 2.**
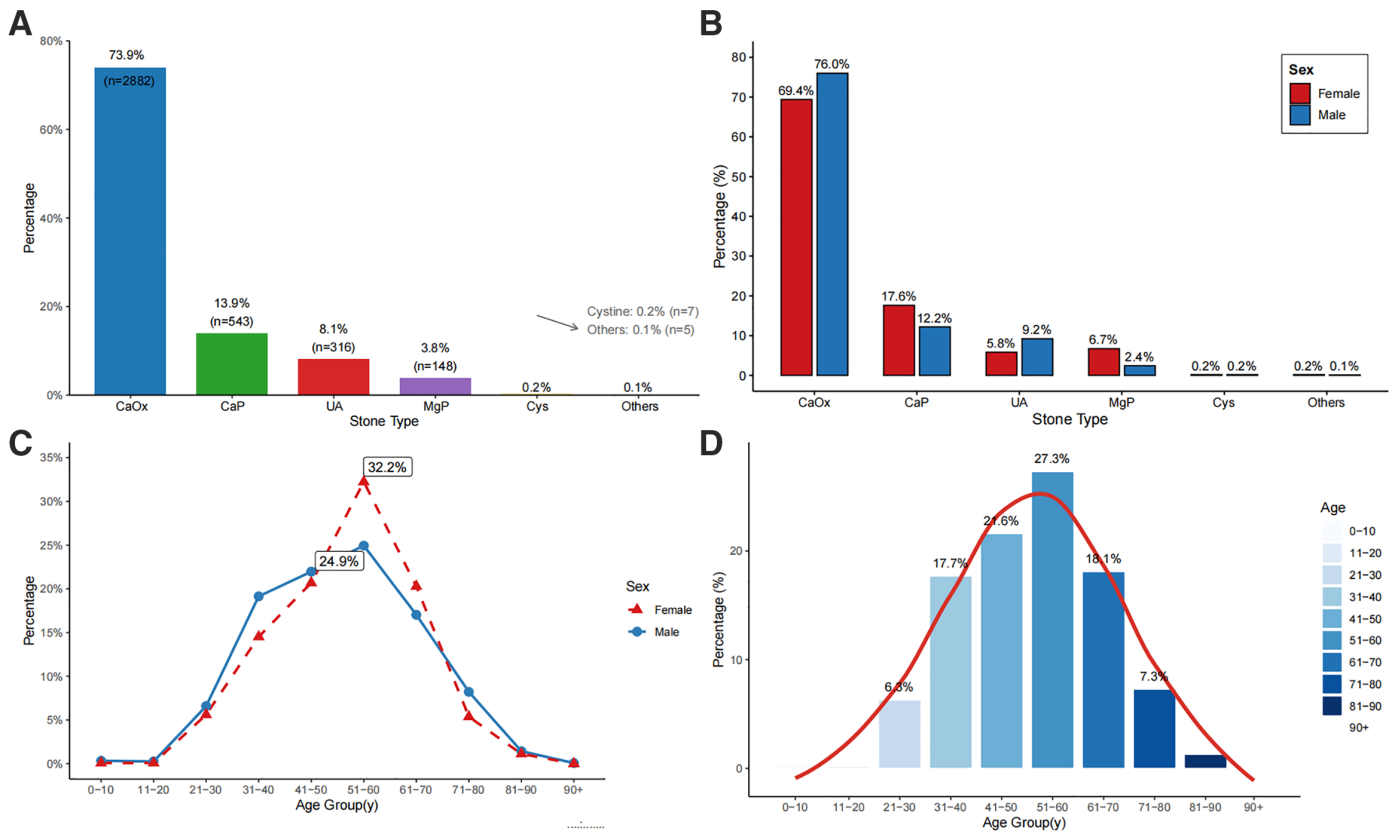
Stone composition and age- and sex-related distributions among patients with urinary stones. A: Overall stone composition in 3901 patients; B: Distribution of stone composition by sex; C: Age distribution stratified by sex; D: Overall age distribution.

A total of 3901 patients with urinary stones were included, with a male predominance (68.0%) and a median age of 52 years (IQR 41–61) (Table 1). Upper urinary tract stones accounted for the majority (88.1%), mainly ureteral (52.0%) and renal stones (36.1%), whereas lower tract stones were less common (11.9%). Mixed stones predominated (82.4%). Under the hierarchical classification, CaOx was the leading primary component (73.9%), followed by CaP (13.9%), UA (8.1%), and MgP (3.8%), while other types were rare (Table 1, Figure 2A).

**Table 1:** Baseline characteristics of 3901 patients with urinary stones.

Characteristic	Characteristic levels	Result
Sex	Male	2654 (68.0%)
	Female	1247 (32.0%)
Age(years)	Range	0 (7 months)-95
	Mean ± SD	51.4 ± 13.9
	Median (IQR)	52.0 (41.0–61.0)
Stone location	upper urinary tract (UUT)	3438 (88.1%)
	Kidney	1408 (36.1%)
	Ureter	2030 (52.0%)
	lower urinary tract (LUT)	463 (11.9%)
	Bladder	385 (9.9%)
	Urethra	78 (2.0%)
Stone composition type (Number of composition)	Pure(1 composition)	687 (17.6%)
	Mixed(2~4 compositions)	3214 (82.4%)
	Mixed(2 compositions)	1795 (46.0%)
	Mixed((3 compositions)	1375 (35.2%)
	Mixed((4 compositions)	44 (1.1%)
Details of stone composition	Calcium oxalate monohydrate	2339 (60.0%)
	Calcium oxalate dihydrate	543 (13.9%)
	Carbonate apatite	476 (12.2%)
	Anhydrous uric acid	272 (7.0%)
	Ammonium urate	38 (1.0%)
	Sodium urate	6 (0.2%)
	Magnesium ammonium phosphate	148 (3.8%)
	Hydroxyapatite	35 (0.9%)
	Calcium hydrogen phosphate	32 (0.8%)
	Cystine	7 (0.2%)
	Calcite	2 (0.1%)
	Xanthine	1 (0.0%)
	Ceftriaxone	2 (0.1%)

### 3.2. Sex-stratified analysis

Stone composition differed significantly by sex (Fig. 2B, Table 2). Male patients had higher proportions of CaOx and UA stones, whereas female patients showed higher proportions of CaP and MgP stones (all *P* < .001). Sex differences were also observed in stone location, with women more likely to present with upper urinary tract stones and men with lower tract stones; these findings are unadjusted and may partly reflect differences in age distribution and referral patterns. Age distributions differed modestly between sexes, but both showed a peak incidence at 51 to 60 years, with most cases occurring between 41 and 70 years (Fig. 2C and D).

**Table 2:** Demographic characteristics, stone location, and stone composition distribution stratified by sex.

Characteristics	Male (N = 2654)	Female (N = 1247)	*P* value
Age (years)			
Median (IQR)	51.0 (40.0–61.0)	53.0 (43.0–61.0)	.007
Age-stratified, n (%)			<.001
0–10	9 (0.3%)	1 (0.1%)	.368
11–20	7 (0.3%)	1 (0.1%)	.499
21–30	175 (6.6%)	70 (5.6%)	.399
31–40	508 (19.1%)	181 (14.5%)	.002
41–50	583 (22.0%)	258 (20.7%)	.499
51–60	662 (24.9%)	402 (32.2%)	<.001
61–70	452 (17.0%)	253 (20.3%)	.034
71–80	218 (8.2%)	67 (5.4%)	.005
81–90	38 (1.4%)	14 (1.1%)	.499
90+	2 (0.1%)	0 (0%)	1
Stone location, n (%)			<.001
Renal stones	872 (32.9%)	536 (43.0%)	<.001
Ureteral stones	1353 (51.0%)	677 (54.3%)	.053
Bladder stones	355 (13.4%)	30 (2.4%)	<.001
Urethral stones	74 (2.8%)	4 (0.3%)	<.001
Urinary tract division, n (%)			<.001
Upper urinary tract (UUT)	2225 (83.8%)	1213 (97.3%)	<.001
Lower urinary tract (LUT)	429 (16.2%)	34 (2.7%)	<.001
Stone composition, n (%)			
CaOx	2016 (76.0%)	866 (69.4%)	<.001
CaP	323 (12.2%)	220 (17.6%)	<.001
UA	244 (9.2%)	72 (5.8%)	<.001
MgP	64 (2.4%)	84 (6.7%)	<.001
Cys	4 (0.2%)	3 (0.2%)	.687
Others	3 (0.1%)	2 (0.2%)	.687

CaOx = calcium oxalate (including calcium oxalate monohydrate and dihydrate), CaP = calcium phosphate (including carbonate apatite, hydroxyapatite, and calcium hydrogen phosphate), Cys = Cystine, IQR = interquartile range, MgP = magnesium ammonium phosphate, UA = uric acid (including anhydrous uric acid, ammonium urate, and sodium urate).

### 3.3. Age-Stratified Analysis

**Figure 3.**
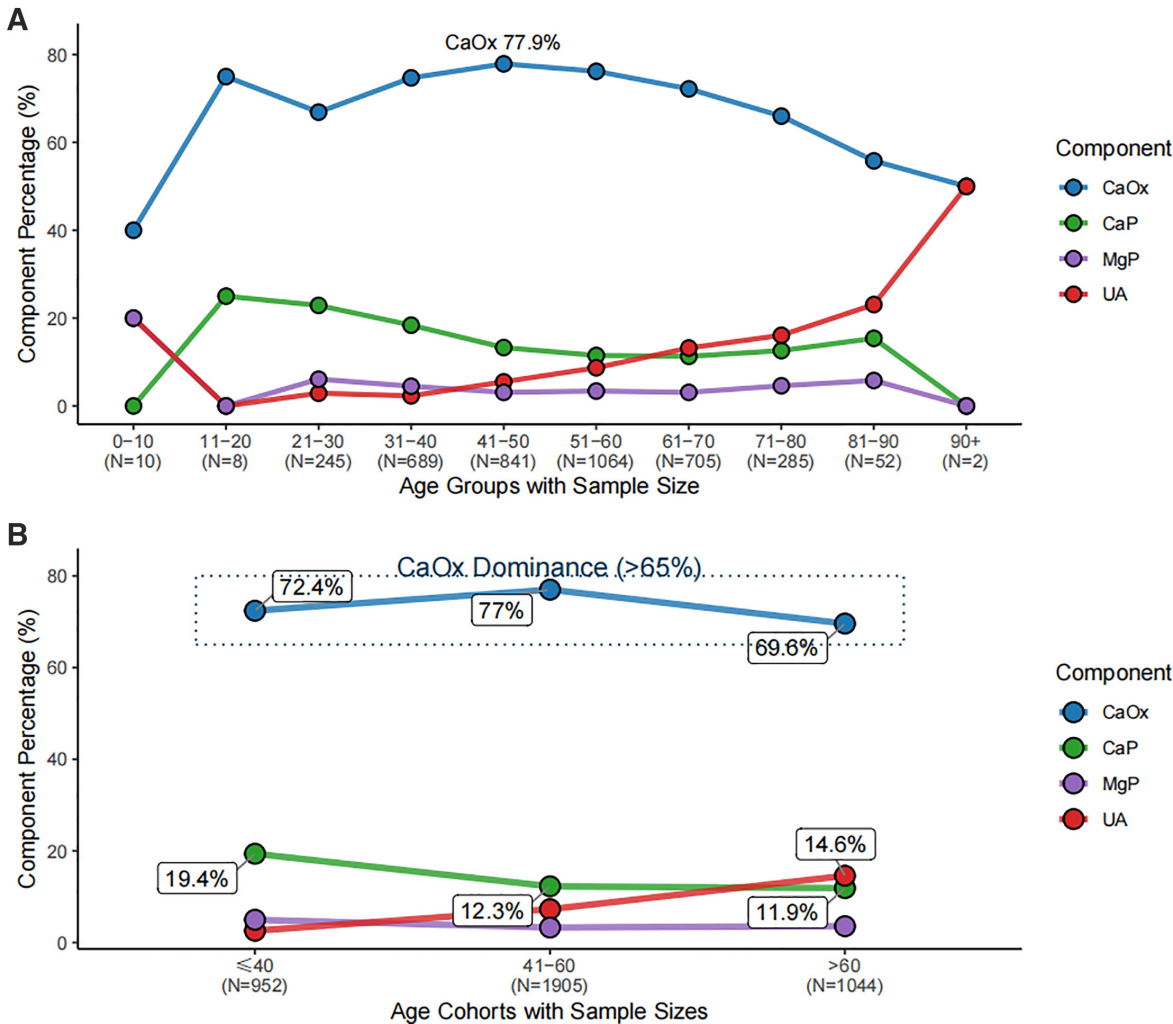
Distribution of stone composition across age groups. A: Proportions of major stone types stratified by 10-year age intervals; B: Proportions of stone types stratified by clinical age groups.

Stone composition varied significantly across age groups (*P* < .001) (Fig. 3A and B, Supplementary Tables S1 and S2). CaOx remained the dominant type but showed a nonlinear pattern, peaking in middle age and declining thereafter. In contrast, UA stones increased markedly with age, whereas CaP stones showed a decreasing trend. Other stone types exhibited relatively minor variation across age groups.

### 3.4. Annual, Monthly, and Seasonal Variation

**Figure 4.**
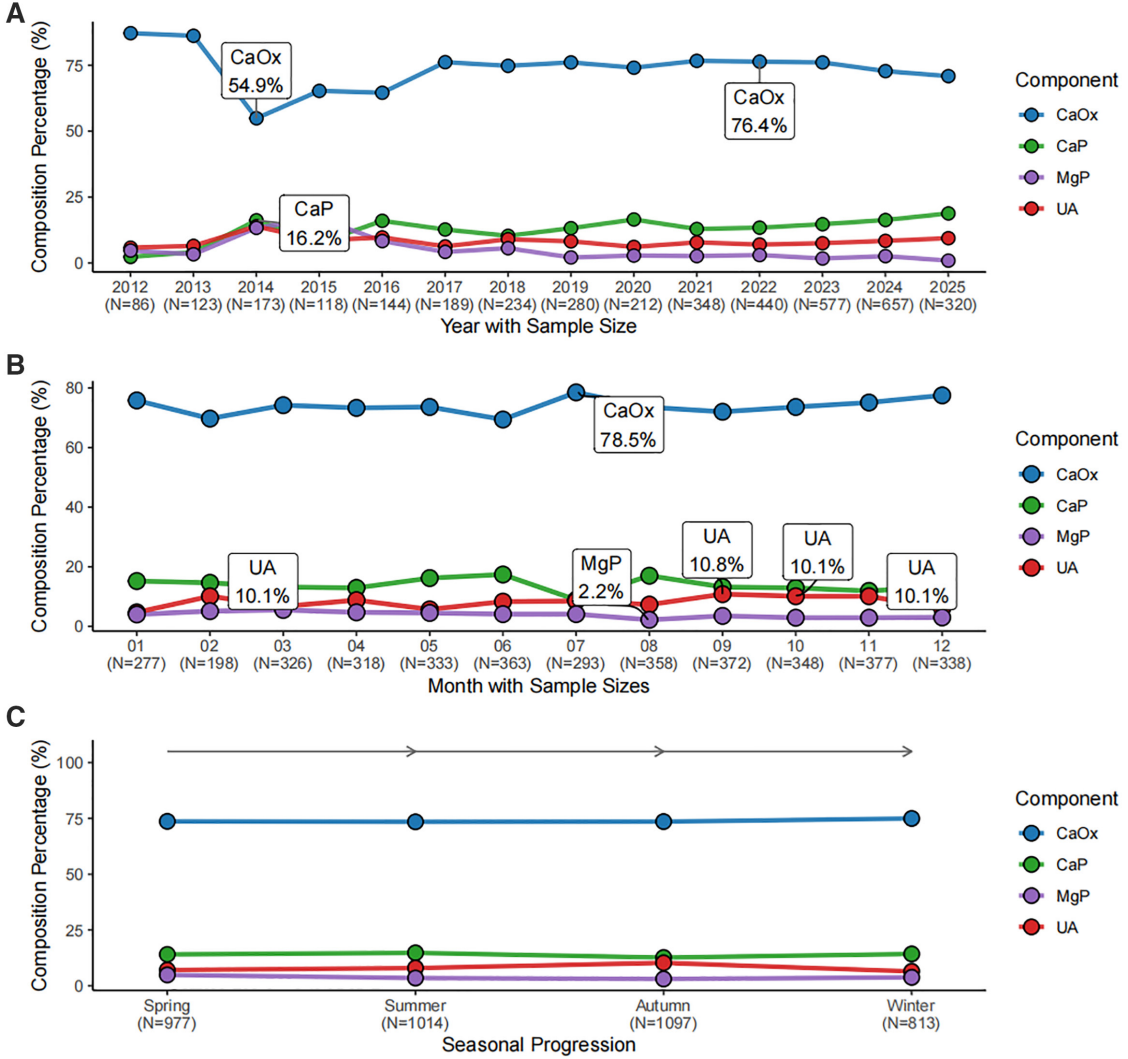
Distribution of stone composition by year, month, and season. A: Stratified by calendar year; B: Stratified by month; C: Stratified by season.

Temporal patterns in stone composition varied across the study period, with fluctuations in CaOx, CaP, and MgP proportions; these patterns are descriptive and may reflect changes in case mix or practice over time (Fig. 4A,Supplementary Table S3).No statistically significant differences were observed across months or seasons (Fig. 4B and 4C, Supplementary Table S4 and S5), although minor fluctuations in CaP and UA proportions were noted.

### 3.5. Associations of Sex and Age With the Risk of Major Stone Types

**Figure 5.**
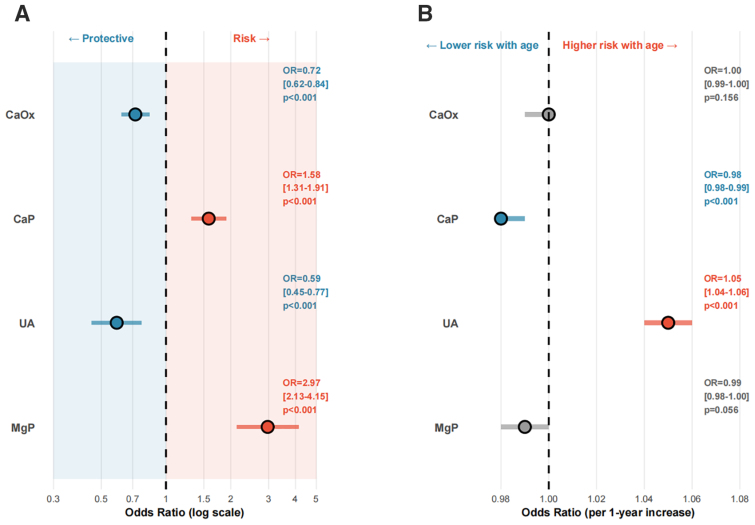
Multivariable logistic regression analysis of factors associated with major stone types. A: Effect of sex (female vs male) on stone type; B: Effect of age (per 1-year increase) on stone type.

**Figure 6.**
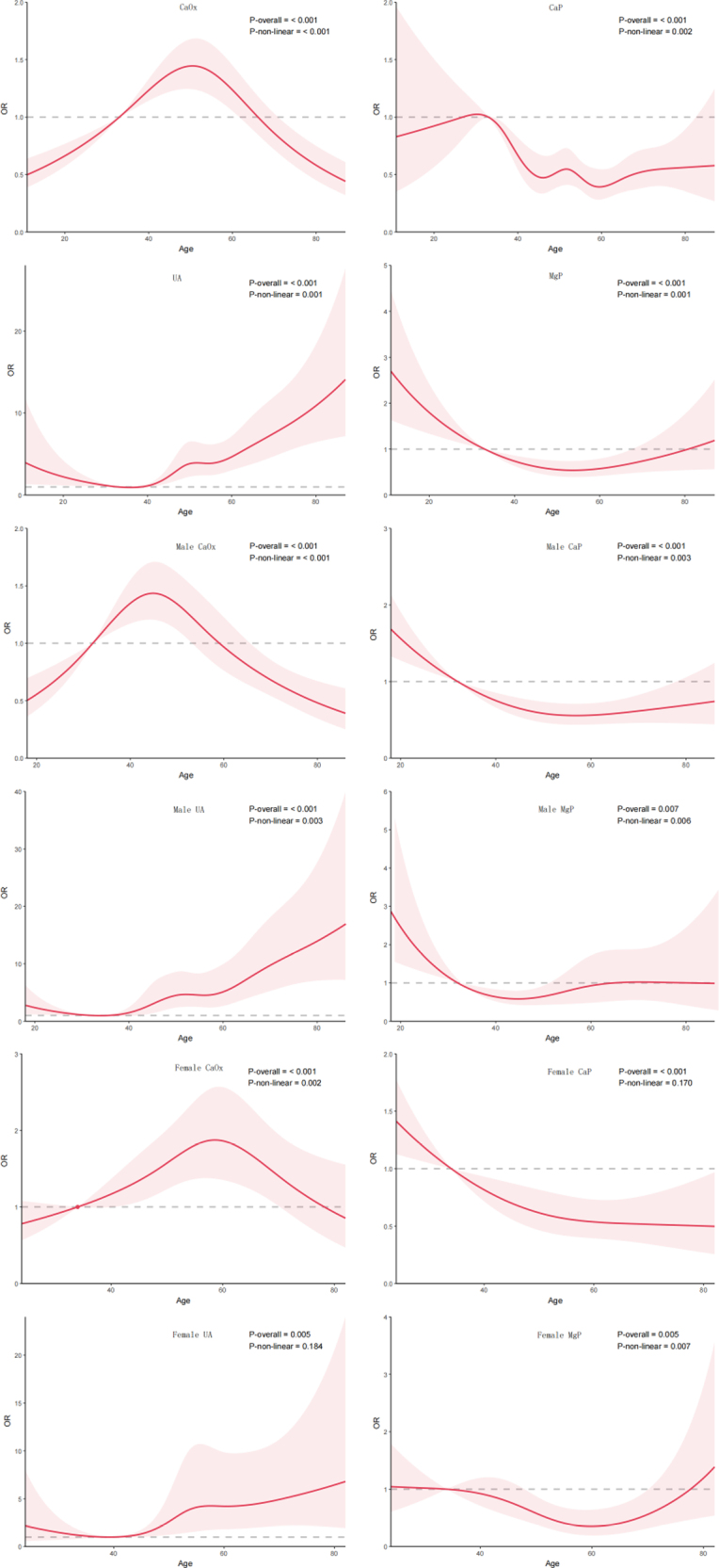
Restricted cubic spline analysis of the relationship between age and the risk of major stone types. The figure shows nonlinear associations between age and stone risk in the overall population and in sex-specific subgroups.

Multivariable analysis showed that sex was independently associated with all major stone types (Fig. 5A), while age was significantly associated with CaP and UA (Fig. 5B). Women had lower odds of CaOx and UA but higher odds of CaP and MgP (all *P* < .001). Increasing age was associated with higher odds of UA and lower odds of CaP.RCS analysis demonstrated nonlinear age–risk relationships (Fig. 6). CaOx showed a typical inverted U-shaped curve in the overall population and in men, with the risk peak approximately at 45–50 years of age. In women, the peak occurred slightly later, at approximately 55–60 years. The risk of CaP generally decreased with advancing age; in men this pattern showed mild nonlinearity, whereas in women it was closer to a linear decline. UA was the only stone type that increased markedly in older age, showing a J-shaped relationship, particularly in men: the odds remained relatively stable before 40 years of age, increased rapidly after 50 years, and reached a high odds ratio at around 80 years relative to age 52; estimates at the extremes of age should be interpreted with caution given wider uncertainty. The age effect of MgP showed clear sex-specific differences: in men, the risk was relatively higher during adolescence and then remained low; in women, the association followed a U-shaped pattern, reached its lowest point at around 60 years, and increased again after 70 years.

## 4. Discussion

Taken together, our 13-year Xiamen cohort paints a coherent picture: CaOx remains the backbone of regional stone disease, yet the overwhelming prevalence of mixed composition signals that single-label summaries can understate biological complexity. The demographic profile—male predominance, median age in the early 50s, and clustering between ages 41 and 70—mirrors large contemporary series^[3–7]^ and anchors the present findings within the broader Chinese literature.^[3,4,9–17]^ Where this report adds texture is in the juxtaposition of stable sex effects, spline-resolved age inflections, and a surprisingly muted seasonal signature in a subtropical coastal setting where climate is frequently invoked as a dominant driver of stone epidemiology.

The higher fraction of mixed stones relative to several northern or inland Chinese cohorts^[10,15,16]^ invites cautious interpretation. Multicomponent stones may reflect sequential or simultaneous changes in urinary supersaturation, urinary pH, and infection milieu rather than a single dominant pathway. Warm, humid coastal living could plausibly promote chronic low-grade dehydration and dietary patterns that differ from those in continental cities, but such hypotheses remain speculative in the absence of paired metabolic phenotyping. Prospective studies that couple FTIR with 24-hour urine chemistries and microbiologic data will be needed to move from correlation to mechanism.

That sex remained an independent determinant of all 4 major stone families – even after multivariable adjustment – strengthens the clinical message that stone prevention should be sex-aware, not merely age-aware. The male skew toward CaOx and UA and the higher proportion of CaP and MgP stones in females align with international experience^[6,11–15,17,18]^ and fit mechanistic narratives involving androgen-related metabolic risk, lower citrate excretion in some male populations, shorter female urethras with higher urinary tract infection burden, and postmenopausal changes in flora and mucosal defenses. Anatomic localization reinforced these themes:97.3% of stones in female patients were located in the upper urinary tract, whereas men contributed disproportionately to bladder and urethral disease – a pattern consistent with prior anatomic descriptions^[3,11,18]^ and compatible with outlet obstruction or voiding inefficiency in men, although we did not capture urodynamic or prostate data to test that link directly.

Age, examined only as a calendar count in routine tables, would have understated the story. RCS modeling revealed why: CaOx risk crests in midlife and tapers thereafter, whereas UA risk accelerates in a J-shaped fashion – most aggressively in men after the sixth decade – echoing observations from France, Iran, and Taiwan.^[8,17,18]^ Renal functional decline, attenuated ammoniagenesis, sustained acidic urine, and cumulative cardiometabolic morbidity offer a biologically plausible scaffold for that late-life UA surge, even if our administrative dataset cannot dissect each pathway. For women, the U-shaped MgP curve – with renewed elevation after age 70 – raises the practical question of whether peri- and postmenopausal patients warrant heightened surveillance for infection, catheter use, and neurogenic bladder comorbidities that predispose to struvite. Conversely, the gradual waning of CaP odds with age, consistent with northern Chinese reports,^[10,14–16]^ suggests that phosphate-dominated crystallization may be more characteristic of younger stone formers in our setting as well.

Temporal analyses revealed a complex pattern. While calendar-year phases hinted at evolving referral, laboratory, or treatment practices, neither month nor season significantly reordered composition – a finding that parallels a northern Chinese single-center report^[10]^ but contrasts with Korean nationwide data.^[5]^ Rather than dismissing either body of work, we favor a contextual reading: in Xiamen maritime climate, seasonal forcing on urine composition may be dampened relative to regions with harsh winters and wide thermal amplitude, where environmental variation is often invoked to explain urolithiasis patterns. That interpretation should not be overextended; subtle monthly trends might emerge with larger samples or individual-level hydration diaries.

Clinically, the data argue against a single uniform prevention strategy. Middle-aged men – where CaOx risk peaks – are rational targets for dietary sodium reduction, adequate fluid intake, and evaluation of hyperoxaluria or enteric risk when indicated. Older men merit deliberate attention to uric acid diathesis, including serum urate monitoring and urine pH-directed alkalinization when appropriate. Women, particularly beyond age 70, should prompt clinicians to think about infection control, anatomic correction when feasible, and closer follow-up after instrumentation. The spline curves are not simply a cosmetic addition; they visualize breakpoints that dichotomous age bands can obscure and may help prioritize counseling intensity at ages where risk curvature is steepest.

We acknowledge important limitations. Single-center tertiary data inherit referral and spectrum bias; missing body mass index, urine pH, cultures, comorbidity granularity, and 24-hour urine panels restrict mechanistic claims; the hierarchical FTIR classification sacrifices secondary mineral signals in mixed stones; absence of recurrence endpoints precludes linking composition to longitudinal outcomes; and secular year effects may partly reflect operational changes in stone submission rather than true epidemiologic shifts. Multicenter prospective designs with standardized metabolic workups will be essential to validate and extend these observations.

## 5. Conclusions

Across more than a decade of FTIR-documented calculi in Xiamen, CaOx-rich stones predominated and mixed composition was the norm, highlighting the need for prevention strategies that embrace biochemical heterogeneity rather than a single “calcium stone” narrative. Sex and age were associated with stone composition in complementary ways – men bearing disproportionate CaOx and UA burden, women facing higher CaP and MgP risk – with spline analyses revealing midlife peaks for CaOx, a late-life surge in UA (especially in men), and a second-phase rise in MgP among older women that should keep infection-related pathways on the differential. Month and season did not significantly restructure composition here, implying that local climate stability may attenuate seasonal effects observed elsewhere and reinforcing the value of individualized over calendar-based risk models. Pending prospective validation, these patterns offer a practical, composition-conscious framework for counseling and secondary prevention in subtropical coastal populations similar to ours.

## Author contributions

**Conceptualization:** Danfeng Sun.

**Data curation:** Wei Yan, Danfeng Sun.

**Formal analysis:** Wei Yan, Danfeng Sun.

**Investigation:** Wei Yan.

**Methodology:** Wei Yan.

**Supervision:** Danfeng Sun.

**Writing – original draft:** Wei Yan.

**Writing – review & editing:** Wei Yan, Danfeng Sun.










